# The role of polysaccharides in immune regulation through gut microbiota: mechanisms and implications

**DOI:** 10.3389/fimmu.2025.1555414

**Published:** 2025-03-31

**Authors:** Ting Zhao, Congyue Wang, Yuhan Liu, Bo Li, Mingjia Shao, Wuyang Zhao, Chuang Zhou

**Affiliations:** ^1^ Department of Oncology, Ansteel Group General Hospital, Anshan, China; ^2^ Department of Medical Oncology, Anshan Cancer Hospital, Anshan, China

**Keywords:** polysaccharides, immune regulation, gut microbiota, SCFAs, cytokines

## Abstract

Polysaccharides, as complex carbohydrates, play a pivotal role in immune modulation and interactions with the gut microbiota. The diverse array of dietary polysaccharides influences gut microbial ecology, impacting immune responses, metabolism, and overall well-being. Despite their recognized benefits, there is limited understanding of the precise mechanisms by which polysaccharides modulate the immune system through the gut microbiota. A comprehensive search of Web of Science, PubMed, Google Scholar, and Embase up to May 2024 was conducted to identify relevant studies. This study employs a systematic approach to explore the interplay between polysaccharides and the gut microbiota, focusing on cytokine-mediated and short-chain fatty acid (SCFA)-mediated pathways. The findings underscore the significant role of polysaccharides in shaping the composition and function of the gut microbiota, thereby influencing immune regulation and metabolic processes. However, further research is necessary to elucidate the detailed molecular mechanisms and translate these findings into clinical applications.

## 1 Introduction

The mammalian gut houses a diverse range of microorganisms, including bacteria, archaea, viruses, fungi, and protozoa, which interact beneficially with the host (1). These gut microbiota influence various processes through the gut-brain axis, affecting metabolism, immune responses, and behavior. With approximately 10¹³ bacteria in the large intestine, they are crucial for immune system development and metabolic capacity (2). The microbiota interact closely with host immunity, distinguishing pathogens from healthy tissues (3). Research shows that short-chain fatty acids (SCFAs) from fiber-rich diets are potential therapies for inflammatory bowel disease and allergic asthma (4). Additionally, disruptions in the microbiota can alter the gut environment, affecting both microbial and host metabolites and compromising immunity (5). Polysaccharides are key carbohydrates involved in energy storage, structural integrity, and immune modulation. Typically well-tolerated, they are found in various foods (6). Plant-derived polysaccharides offer diverse health benefits, including immune regulation, anti-inflammatory effects, antiviral capabilities, antitumor effects, and hypoglycemic potential, all linked to their structural attributes (7). These polysaccharides influence the gut microbiota, with dietary polysaccharides being broken down by specialized enzymes from the gut microbiome (8). A diet rich in polysaccharides supports beneficial gut microbes and enhances overall well-being. For example, inulin can reduce endotoxins and stimulate gut bacteria growth (9). Recent studies suggest that polysaccharides may modulate immune responses and help manage conditions like SARS-CoV-2 infection (10). This study focuses on the effects of polysaccharides on gut microbes and immune regulation, specifically through cytokine and SCFA pathways.

## 2 Properties of polysaccharides

Polysaccharides exhibit a wide range of structural and functional properties that significantly influence their interactions with the gut microbiota and the immune system. This section examines polysaccharides derived from plants, algae, and fungi, highlighting their structural characteristics and biological activities.

### 2.1 Plant-derived polysaccharides

Plants are a primary source of polysaccharides, which play essential roles in both structure and function. Polysaccharides like pectin, found in plant cell walls, and homogalacturonans, composed of α-(1–4)-linked D-galacturonic acid, are particularly important. Cellulose, a major component of green plants and algae, consists of β(1→4)-linked D-glucose units. Insulins, mainly composed of fructose units linked by β(2→1) glycosidic bonds, are commonly found in various plants. Plant polysaccharides have been shown to impact health conditions such as diabetes and liver fibrosis. Liu et al. (11) found that polysaccharides from Dendrobium officinale (DOP) enhance glycogen synthesis and stability, inhibit glycogen degradation, and suppress gluconeogenesis. Wang et al. (12) demonstrated that DOP reduces inflammation by inhibiting LPS/TLR4/NF-κB signaling, decreasing TGF-β and TNF-α levels, lowering collagen I and α-SMA expression, and increasing IL-10 expression.

### 2.2 Algae-derived polysaccharides

Algae, unique among photosynthetic organisms, possess polysaccharides that resist human digestive enzymes but can be fermented by gut bacteria. These polysaccharides, including sulfated types such as carrageenans, agars, porphyrin, and xylan found in red algae, exhibit notable biological properties. They are particularly significant for their anticoagulant effects, which help prevent thrombus formation and reduce the risk of clotting. Tang et al. (13) highlighted the anticoagulant and antioxidant properties of polysaccharides from Grateloupia livida. Jia et al. (14) found that algal polysaccharides positively affect blood glucose, triglycerides, cholesterol, alanine transaminase, and blood urea nitrogen levels in type 2 diabetic rats, indicating their potential as natural pharmaceuticals.

### 2.3 Fungi-derived polysaccharides

Edible fungi contain a variety of polysaccharides, including heteropolysaccharides and glucans, which exhibit biological activities such as immunomodulation, antioxidation, and potential health benefits. These polysaccharides enhance the nutritional value of edible fungi and hold promise for functional foods and natural medicines. Observational studies have shown their roles in addressing obesity, immunity, and cognitive function. Chang et al. (15) noted that high molecular weight polysaccharides from Ganoderma lucidum may improve gut health and help manage metabolic disorders linked to obesity. Luo et al. (16) found that polysaccharides from Ganoderma lucidum may alleviate colorectal cancer by influencing gut bacteria and gene expression. Poria cocos polysaccharides, mainly (1→3)-β-glucans with (1→6)-β-glucose side chains, are key bioactive components with properties such as cancer-fighting, immunomodulation, anti-inflammatory, anti-aging, antioxidant, anti-diabetes, and anti-hemorrhagic fever effects (6).

## 3 Polysaccharides and gut microbiota interaction

**Figure 1:**
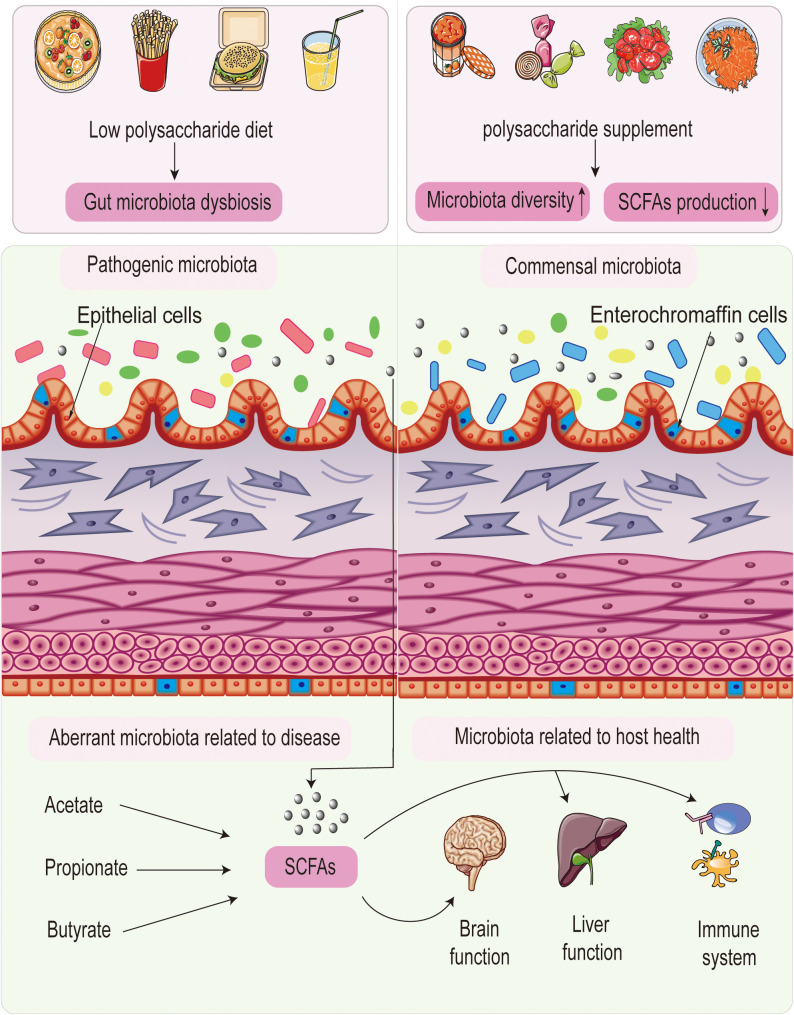
Interaction between polysaccharides and gut microbiota.

Polysaccharides play a crucial role in shaping the gut microbiota, providing probiotics with a competitive advantage over pathogenic bacteria. Additionally, the gut microbiota are significantly influenced by polysaccharides with various chemical structures. Together, polysaccharides function as prebiotics, helping to regulate the microecological environment of the gut. **Figure 1** illustrates the interaction between polysaccharides and gut microbiota.

### 3.1 The variability of gut microbiota

Evidence suggests that the composition of gut microbiota is closely linked to polysaccharide intake. Thomson et al. (17) demonstrated that co-cultures of Bacteroides dorei and Clostridium symbiosum grew rapidly on xylan, leading to a reduction in inflammation. Population studies have shown that barley β-glucan increases the abundance of Bacteroides and Bifidobacteria in older adults, highlighting its prebiotic potential (18). *In vitro* studies by Yang et al. (19) found that pectin, inulin, and β-glucan promoted the proliferation of Bifidobacteria and Erysipelotrichaceae. Shang et al. (20) proposed that fucoidans may help improve intestinal dysbiosis by increasing Akkermansia, potentially alleviating metabolic syndrome.

### 3.2 The production of advantageous metabolites

The symbiotic gut microbiota harnesses energy and produces a wide array of molecules and metabolites, including key SCFAs such as acetate, propionate, and butyrate (21–23). SCFAs exert their effects through various mechanisms, including acidifying the intestinal environment and maintaining intestinal barrier function. For instance, SCFAs play a significant role in mammalian energy metabolism by facilitating the fermentation of dietary fiber by anaerobic bacteria in the gut (24). Another study found higher concentrations of SCFAs in the proximal colon, where enterocytes either utilize them locally or transport them across the intestinal epithelium (25). These findings highlight the importance of SCFAs in maintaining gut and immune homeostasis. Acetate, a common anion in biological systems, is utilized by organisms as acetyl coenzyme A, a critical substrate. Intraperitoneal administration of sodium acetate at varying doses has been observed to trigger headaches in sensitized rats. It is postulated that acetate, a byproduct of ethanol oxidation, contributes to the manifestation of hangover symptoms. Elevated serum acetate levels lead to adenosine accumulation in various tissues, including the brain. Interestingly, post-ethanol treatment with the adenosine receptor antagonist caffeine was found to alleviate nociceptive responses in rat models. Furthermore, acetate has well-documented immunomodulatory properties, impacting the innate immune response against pathogens like Haemophilus influenzae, a major respiratory pathogen. Meanwhile, butyrate has emerged as a key regulator in immune homeostasis, acting locally in the gastrointestinal tract and systemically through circulation. By inhibiting class I histone deacetylases and stimulating G-protein coupled receptors such as GPR109A, GPR43, and GPR41, it exerts immunomodulatory effects (26). Among the various SCFAs, butyrate uniquely serves as a ligand for HCA2 in intestinal regulatory T cells in experimental settings (27). Its critical role in modulating colonic inflammation has made it a potential agent for both preventing and treating inflammation-related conditions like ulcerative colitis and colorectal cancer.

It is the intricate enzymatic machinery of the intestinal microbiota that generates SCFAs from polysaccharides (21). Noteworthy microbial inhabitants are involved in SCFA synthesis in response to polysaccharide stimuli. For instance, the Wood-Ljungdahl pathway utilized by Blautia hydrogenotrophica facilitates acetate production within this metabolic framework (28). Propionate biosynthesis occurs via the succinate pathway within the Bacteroidetes phylum, which includes taxa such as Lachnospiraceae and Negativicutes (29). Metagenomic analyses highlight that gut microbiota species are crucial to butyrate production, with specific subsets of Lachnospiraceae capable of converting lactate and acetate into butyrate (30). Furthermore, Akkermansia plays a significant role in converting mucin into SCFAs, adding another layer of complexity to the gut ecosystem’s metabolic interactions (20). Research suggests that fucoidan supplements can promote the proliferation of specific microbes, enhancing SCFA production. *In vitro* and animal studies further support this, showing significant increases in SCFA synthesis with high barley β-glucan intake (18). Interventions involving plant-derived polysaccharides, in combination with cross-feeding mechanisms between lactate-producing bacteria, likely contribute to maintaining a well-functioning microbial community capable of producing butyrate (31). Additionally, Astragalus membranaceus polysaccharides have been shown to modulate SCFA composition and increase the Bacteroidetes-to-Firmicutes ratio (a marker of gut dysbiosis) in diabetic mice (32).

### 3.3 Mechanisms by which different structures of polysaccharides regulate immune responses via specific receptors or pathways

Polysaccharides, as complex carbohydrates, regulate immune responses by interacting with specific receptors on host cells (33). The molecular weight, degree of branching, and functional groups of polysaccharides influence their immunomodulatory mechanisms. Below are the mechanisms by which different structural polysaccharides regulate immune responses through specific receptors or pathways:

(1) β-Glucans: β-Glucans, commonly found in fungi, yeast, and algae, primarily modulate immune responses via pattern recognition receptors (PRRs). Specifically, Dectin-1 is the main receptor for β-glucans. Binding of β-glucans to Dectin-1 activates the Syk signaling pathway, which in turn triggers NF-κB activation, promoting the production of pro-inflammatory cytokines such as TNF-α and IL-6 (34). Additionally, β-glucans can activate macrophages through Toll-like receptor (TLR) 2, enhancing their phagocytic capabilities and strengthening the host’s defense against pathogens. (2) Arabinogalactan: Arabinogalactan, abundant in plants, particularly in traditional medicinal herbs like Astragalus membranaceus, primarily activates immune responses by modulating TLR4 (35). Upon binding to TLR4, arabinogalactan activates the MyD88-dependent signaling pathway, inducing the secretion of IL-12 and promoting T cell polarization and activation. (3) Pectins: Pectins, complex polysaccharides derived from plant cell walls, possess highly branched structures. Their immunomodulatory effects are primarily mediated through interactions with Toll-like receptors (TLR2 and TLR4) on macrophages. Studies have shown that pectins can inhibit the NF-κB pathway, reducing the production of pro-inflammatory cytokines and exerting anti-inflammatory effects (36). Furthermore, pectins can interact with C-type lectin receptors (CLRs), enhancing antigen presentation and adaptive immune responses. (4) Sulfated Polysaccharides: Sulfated polysaccharides, such as carrageenan from seaweed, exert immunomodulatory effects closely linked to their sulfate groups. They interact with C-type lectin receptors (e.g., DC-SIGN) to regulate dendritic cell and T cell functions (37). Additionally, sulfated polysaccharides can inhibit complement activation, reducing inflammatory responses.

### 3.4 Differential roles of short-chain fatty acids in immune regulation

SCFAs, including acetate, propionate, and butyrate, are metabolic products produced by gut microbiota through the fermentation of dietary fibers. These SCFAs play critical roles in immune regulation, with each fatty acid modulating the immune system through distinct mechanisms. (1) Acetate: Acetate is one of the most abundant SCFAs and plays multiple roles in gut immunity. It strengthens the intestinal mucosal barrier, preventing pathogen invasion. Acetate binds to G-protein coupled receptors, particularly GPR43, which activates anti-inflammatory pathways, including the promotion of IL-10 production. IL-10 is a key regulatory cytokine that controls immune responses and prevents excessive inflammation (38). Additionally, acetate suppresses the production of pro-inflammatory cytokines, exerting systemic anti-inflammatory effects. Through these mechanisms, acetate plays a vital role in maintaining intestinal homeostasis and overall immune balance. (2) Propionate: Propionate primarily influences immune function by modulating metabolic pathways. Like acetate, propionate interacts with GPR43 and GPR41 receptors to regulate gut immune responses (39). A critical function of propionate is the promotion of regulatory T cell (Treg) generation. Tregs are specialized immune cells that suppress inflammatory responses and maintain immune tolerance (40). By enhancing Treg activity, propionate helps reduce inflammation, particularly in chronic inflammatory conditions. Additionally, propionate influences fat and glucose metabolism, indirectly affecting immune function through metabolic regulation. (3) Butyrate: Butyrate serves as the primary energy source for colonocytes and regulates immune function through several mechanisms (41). One key mechanism is the inhibition of histone deacetylases (HDACs), which modulates gene expression in immune cells. This action not only promotes the generation of regulatory T cells but also suppresses the release of pro-inflammatory cytokines, reducing inflammation. Butyrate has demonstrated significant effects in preventing and treating inflammatory bowel diseases (IBDs) such as ulcerative colitis and Crohn’s disease (42). By maintaining the integrity of the intestinal epithelial barrier and reducing inflammation, butyrate aids in alleviating disease symptoms and promoting gut healing.

## 4 Polysaccharide pathogenesis

**Figure 2:**
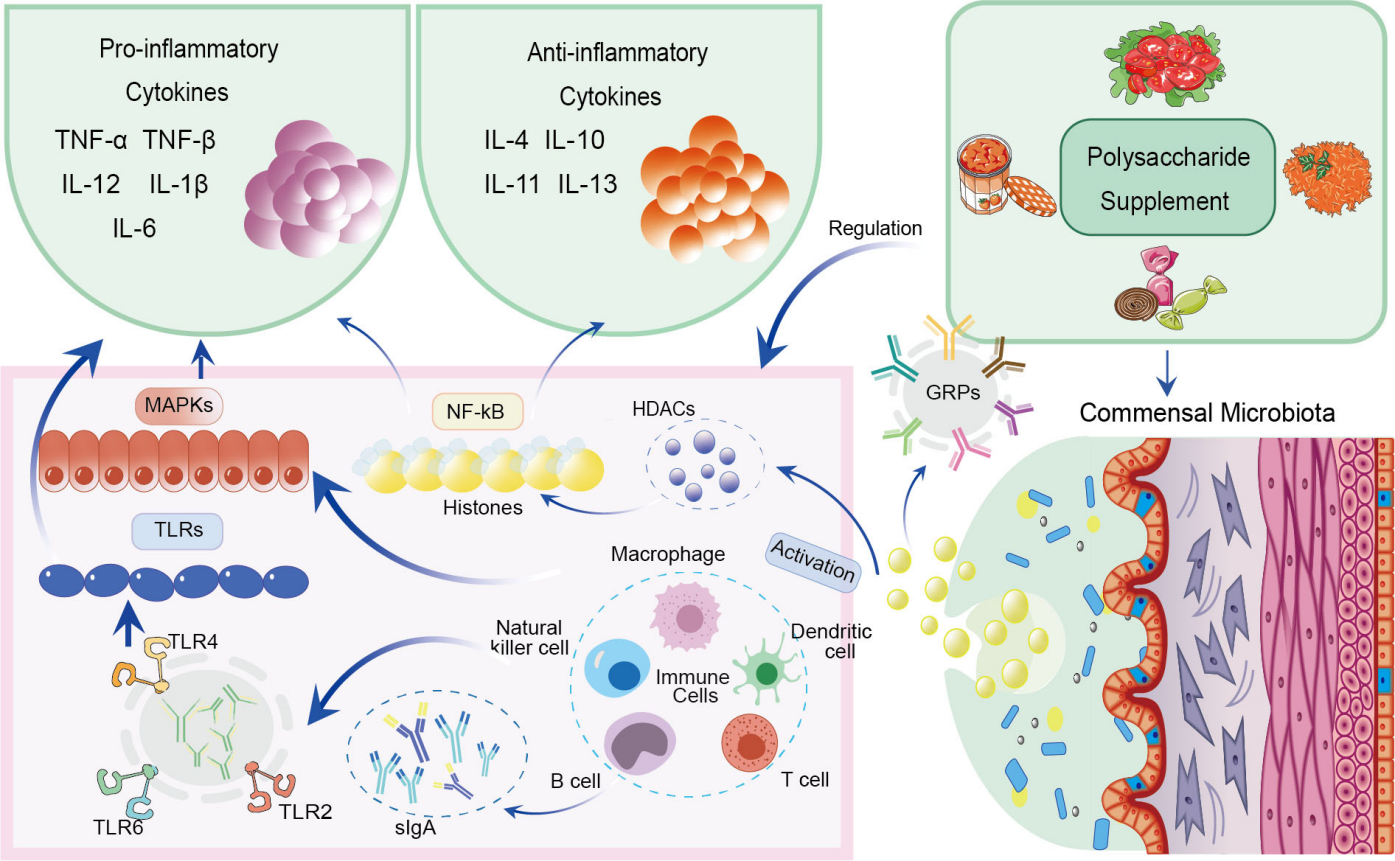
Immune regulation mechanisms mediated by polysaccharides via cytokines and SCFAs.

A summary of the immune regulation mechanisms mediated by cytokines and SCFAs is illustrated in **Figure 2**.

### 4.1 Stimulation of immune cells by cytokines

A diverse array of immune cells, including macrophages, B lymphocytes, T lymphocytes, mast cells, endothelial cells, fibroblasts, and others, produce cytokines, which are essential signaling molecules (43). These cytokines exert their effects through cell surface receptors and play crucial roles in both pro-inflammatory and anti-inflammatory pathways. Additionally, cytokines are being explored as potential therapeutic targets for alleviating pathological pain resulting from inflammation or peripheral nerve injury (44). Furthermore, the modulation of pro-inflammatory cytokines by polysaccharides is being investigated as a strategy to mitigate immunosuppressive effects in patients undergoing treatment for immune-related conditions. In contrast, anti-inflammatory cytokines play regulatory roles in dampening inflammatory responses and balancing the actions of pro-inflammatory cytokines (45). Key cytokines that influence immune system function include IL-4, IL-10, IL-11, and IL-13.

Macrophages are key players in initiating various immune responses. Polysaccharides have been shown to activate macrophages, influencing cytokine secretion through diverse signaling pathways. Gupta et al. (46) demonstrated that polysaccharides extracted from Tinospora cordifolia (TCPs) can enhance the expression of nitric oxide synthase (NOS2), leading to increased nitric oxide production and enhanced microbicidal capabilities of macrophages. However, certain polysaccharides have immunosuppressive properties, inhibiting NF-κB and MAPK-mediated inflammation triggered by lipopolysaccharides (LPS). T cells consist of various helper subsets, each with distinct functions mediated by the secretion of specific cytokine profiles. Chen et al. (47) revealed that, through its interaction with Toll-like receptors, YCP serves as a secondary signal that activates T cells, promotes proliferation, and increases IFN production. Furthermore, by activating TLR-4 on dendritic cells, YCP promotes IL-12 secretion and upregulates CD80, CD86, and MHC II markers (48). Activated T cells are crucial for promoting the secretion of IFN-γ and IL-12 while suppressing IL-4 production, a mechanism vital for enhancing immune responses to foreign antigens (49). In contrast, immunoglobulins produced by B cells play a significant role in immune defense against exogenous antigens (50). Additionally, microglia, the innate immune cells of the central nervous system, produce various inflammatory cytokines in response to injury or infection.

### 4.2 Immune modulation and SCFA-induced polysaccharides

In the context of gut microbiota-driven anaerobic fermentation, the breakdown of complex polysaccharides generates a diverse array of microbiota-derived metabolites. Among these, SCFAs are key modulators of immune function. Growing evidence highlights the ongoing regulatory influence of SCFAs on both local and systemic immune homeostasis, as well as immune cell functionality (51). SCFAs exert their effects through the inhibition of histone deacetylases and the activation of G protein-coupled receptors (GPCRs), which confer beneficial effects on immune cells. In a study by Chang et al. (52), the bacterial metabolite n-butyrate was shown to modulate intestinal macrophages by inhibiting histone deacetylases, making them less responsive to commensal microbes in the colon. SCFAs also play a crucial role in promoting the generation of anti-inflammatory cytokines. They activate the inflammasome in immune cells through a GPCR-mediated mechanism, involving hyperpolarization and calcium mobilization (53). This pathway leads to the synthesis of IL-10, a key anti-inflammatory cytokine, which depends on the suppression of histone deacetylase activity and activation of GPR43. Moreover, Ouyang et al. (54) emphasized the critical role of IL-22 in maintaining intestinal barrier integrity, balancing intestinal equilibrium, and protecting against inflammation. Recent studies have shown that innate lymphoid cells and CD4^+^ T cells produce IL-22 in response to SCFAs through the activation of GPR41 and inhibition of histone deacetylases (55).

## 5 Structural features of polysaccharides

The conformation of polysaccharides plays a significant role in their interaction with the immune system. For example, Satitmanwiwat et al. (56) found that triple-helix conformations exhibit greater immunomodulatory efficacy compared to β-glucans, as they stimulate TNF-α release by immune cells. The molecular weight of polysaccharides also influences immune responses, with higher molecular weight polysaccharides generally showing stronger effects. Zhao et al. (57) demonstrated that the immune effects of Schisandra chinensis polysaccharides are inversely related to their molecular weight. On the other hand, Chen et al. (58) found that lower molecular weight polysaccharides, with simpler structures, are more likely to cross cellular barriers. Functional groups in polysaccharides also impact immune responses in distinct ways. For instance, higher acetylation levels may reduce immunomodulatory effectiveness (59), while sulfate groups in pectic polysaccharides can enhance macrophage and neutrophil function, potentially improving immune responses (60). Additionally, branching in polysaccharides—driven by monosaccharide residues or chains—can affect immune regulation, such as IL-6 secretion induced by LPS (61). The details of these relationships are summarized in **Table 1**. Despite these insights, further research is needed to fully understand the structure-function relationships in immune-regulating polysaccharides and explore their potential applications in functional foods.

**Table 1:** Structural features and therapeutic applications of polysaccharides in immune-related diseases.

Polysaccharide Type	Source	Key Structural Features	Immune Mechanisms	Disease Applications
β-Glucans	Fungi (e.g., Ganoderma lucidum)	β(1→3)/(1→6) linkages	Dectin-1/Syk/NF-κB activation; Macrophage polarization	Colorectal cancer, Allergic asthma
Fucoidan	Brown algae	Sulfated L-fucose residues	TLR4 inhibition; Treg induction	Rheumatoid arthritis, IBD
Inulin	Chicory root	β(2→1)-fructans	Bifidobacteria proliferation; SCFA production	Type 1 diabetes, Melanoma
Pectin	Citrus peel	α(1→4)-galacturonic acid	TLR2/4 modulation; IL-10 upregulation	Inflammatory bowel disease

## 6 The current understanding of SCFA and the immune system

Bacteria ferment indigestible dietary fibers anaerobically to produce SCFAs, including acetate, butyrate, and propionate. These SCFAs can influence systemic health by signaling through G-protein-coupled receptors, such as GPR41, GPR43, and GPR109a (62, 63). They play a crucial role in regulating gut homeostasis and maintaining epithelial barriers. Butyrate, for example, acts as an inhibitor of histone deacetylase, promoting colonocyte proliferation, inhibiting stem cell proliferation, and regulating the polarization of anti-inflammatory macrophages (64). Additionally, butyrate directly stimulates colonic mucus secretion by upregulating Muc2 and glycoltransferase expression (65) and indirectly through autophagy. SCFAs also provide anti-inflammatory protection by regulating Tregs (66). High-fiber diets protect against allergies in mice by impairing Th2 differentiation and enhancing dendritic cell phagocytosis, whereas low-fiber diets exacerbate allergic inflammation (67). In type 1 diabetes, increased SCFA levels are linked to improved symptoms, which may result from a reduction in autoreactive T cells, induction of Tregs, and enhanced gut barrier function (68). It has been proposed that SCFAs can exert beneficial effects by directly influencing gut-derived hormones like GLP-1 and peptide YY, in addition to impacting systemic circulation (69). Beyond metabolic benefits, SCFAs stimulate intestinal gluconeogenesis (70). When administered alone or in combination—whether through drinking water, nanoparticles, or acetate supplementation—propionate, butyrate, and acetate can improve host metabolism and physiology (71). Notably, the effects of propionate are believed to be mediated through peripheral nerve stimulation of the vagus nerve. In contrast, high-fat diets increase gut microbiota-mediated acetate turnover, which promotes hyperphagia by increasing ghrelin secretion and enhancing energy storage (72). These effects appear to depend on the specific site where SCFAs are stimulated, emphasizing the need for further research into their role in regulating obesity.

Despite the growing interest in understanding the interaction between SCFAs and the immune system, several knowledge gaps remain. More research is needed to elucidate the molecular mechanisms by which SCFAs—such as acetate, butyrate, and propionate—affect immune cells, including Tregs, macrophages, and dendritic cells. The context-dependent effects of SCFAs in diseases like inflammatory bowel disease, type 2 diabetes, and allergic asthma also require further investigation to fully realize their therapeutic potential (73). Additionally, the role of SCFAs in the gut-brain axis, personalized nutrition, and long-term health impacts warrants deeper exploration. Developing innovative therapeutic formulations aimed at modulating SCFA levels, as well as understanding their interactions with other gut-derived metabolites, are promising yet underexplored areas. Finally, further research is needed to explore the relationship between SCFAs and obesity, as well as other metabolic disorders, including their feedback mechanisms with the nervous system. Addressing these gaps may lead to the development of novel therapeutic strategies for treating immune-related and metabolic diseases.

## 7 Prospects

Several critical areas for further exploration regarding the interaction between SCFAs and the immune system include understanding the molecular mechanisms through which SCFAs modulate immune cell functions. This knowledge could lead to therapeutic applications for autoimmune and inflammatory diseases. Additionally, investigating the role of SCFAs in diseases such as inflammatory bowel disease, type 2 diabetes, and allergic asthma is essential to determine how SCFAs influence disease progression and treatment outcomes. The gut-brain axis represents another frontier, particularly in understanding how SCFAs affect neurological diseases and behaviors through mechanisms like inflammation, appetite regulation, and neural signaling. Personalized nutrition, tailored to an individual’s microbiome and SCFA profile, could optimize health outcomes. However, the long-term effects of sustained SCFA modulation via diet or supplementation remain unclear, highlighting the need for longitudinal studies. Developing therapeutic formulations to modulate SCFA levels or mimic their effects offers exciting new treatment possibilities. Moreover, understanding the interactions between SCFAs and other metabolites, such as bile acids and amino acid derivatives, is crucial for a more comprehensive understanding of their roles in health and disease.

Recent studies highlight the therapeutic potential of polysaccharide prebiotics in cancer and autoimmune disorders. For instance, fucoidan in colorectal cancer patients enhances chemotherapy sensitivity by upregulating Akkermansia abundance and suppressing TLR4/NF-κB signaling (74). In rheumatoid arthritis, β-glucan activates Dectin-1 to promote Treg differentiation and reduce pro-inflammatory cytokines (e.g., IL-17, TNF-α) (75). A clinical trial (NCT04128072) demonstrated that inulin combined with immune checkpoint inhibitors improved melanoma patients’ ORR (45% vs 28%) (76). Future research should focus on dose optimization and targeted delivery strategies for clinical translation.

## 8 Conclusion

The gut microbiota are well-known for their role in chronic autoimmune diseases, particularly brain-related disorders. These conditions involve complex signaling pathways that include neurotransmitters, immune cells, and microbiota-derived metabolites. While manipulating the microbiota to treat diseases such as multiple sclerosis (MS) shows promise, more systematic research is needed. Dietary interventions, including polysaccharides, have the potential to enhance immunity by regulating gut microbiota. Polysaccharides exhibit immunomodulatory and prebiotic effects, although their use in boosting immunity is not yet widespread. Future research should focus on understanding how these substances balance immune enhancement and immunosuppression, as well as determining specific intake patterns to modulate pro-inflammatory cytokines. Additionally, further investigation is needed to better understand the mechanisms promoting MS.
